# Cutoff criteria for the placebo response: a cluster and machine learning analysis of placebo analgesia

**DOI:** 10.1038/s41598-021-98874-0

**Published:** 2021-09-28

**Authors:** Per M. Aslaksen

**Affiliations:** Department of Psychology, The Faculty of Health Sciences, UiT The Arctic University of Norway, 9037 Tromsø, Norway

**Keywords:** Psychology, Signs and symptoms, Pain

## Abstract

Computations of placebo effects are essential in randomized controlled trials (RCTs) for separating the specific effects of treatments from unspecific effects associated with the therapeutic intervention. Thus, the identification of placebo responders is important for testing the efficacy of treatments and drugs. The present study uses data from an experimental study on placebo analgesia to suggest a statistical procedure to separate placebo responders from nonresponders and suggests cutoff values for when responses to placebo treatment are large enough to be separated from reported symptom changes in a no-treatment condition. Unsupervised cluster analysis was used to classify responders and nonresponders, and logistic regression implemented in machine learning was used to obtain cutoff values for placebo analgesic responses. The results showed that placebo responders can be statistically separated from nonresponders by cluster analysis and machine learning classification, and this procedure is potentially useful in other fields for the identification of responders to a treatment.

## Introduction

The use of placebos is crucial in medical science to determine the effects and efficacy of treatments^1,2^. Because randomized controlled trials (RCTs) use placebo conditions as a control, the definition of placebo responders is important to make valid statements about the statistical effects of treatments^3^. However, there is no consensus on how to statistically define a placebo responder or how to determine the cutoff for when a placebo response is large enough to be considered a true change from baseline. The majority of studies investigating the mechanistic components of the placebo response have used the placebo response in the context of pain, i.e., placebo analgesia, as the modality for studying this effect^4^.

Experimental studies on the mechanisms of the placebo response have traditionally relied on defining a placebo effect as the statistically significant difference in mean values between a group that receive placebo treatment and a group that receive no treatment but undergo the same assessments and procedures as the placebo group^5^. However, statistically significant differences are largely dependent on sample sizes, and larger samples might produce significant differences between groups that may have no practical or clinical relevance^6^. In the field of clinical pain, several authors have previously addressed this issue, and values for minimal clinically important differences in pain change have been suggested in terms of absolute changes and percentage changes. In a study on acute pain in trauma patients, Todd et al.^7^ found that an absolute pain reduction of 13 mm on a 100-mm visual analog scale (VAS) was a valid cutoff value for a clinically significant change in acute pain. The finding by Todd et al. was replicated in other studies^8,9^, but there was the notion that patients with higher pain at baseline required a higher VAS reduction compared to those with lower initial VAS ratings to achieve a meaningful reduction in pain^9^. The relative percentage change in pain might in some situations be less biased by baseline or pretreatment pain compared to the absolute VAS change, and Jensen et al.^10^ suggested that more than a 33% relative and 20–30 mm absolute reduction in individual VAS scores constituted a clinically meaningful change in postoperative pain. In a study employing meta-regression on data from 10,938 patients from 40 studies with various diagnoses of chronic pain, a minimum clinically important difference of 28 mm on the 100-mm VAS scale was found^11^. Thus, the cutoff values for a clinically relevant VAS change vary among acute, postoperative, and chronic types of pain.

However, most mechanistic studies on placebo analgesia have been performed in healthy volunteers who had no prior history of long-lasting pain. Thus, the most comparable clinical category is probably acute pain. By using the cutoff value from Todd et al.^7^, a meta-analysis on placebo effects^12^ found that only 26% of the included placebo studies performed in healthy volunteers reported clinically significant pain reduction after placebo treatment. Minor pain reductions in placebo analgesic studies could be problematic for the interpretation and generalizability of mechanistic studies on the placebo response. Furthermore, the reported response rate to placebo treatment varies substantially between studies and between experimental and clinical settings^13,14^. In an influential and debated study by Beecher^15^, the response rate to placebo in clinical pain was estimated to be 35.2%, whereas other more recent studies have shown values between 39–78%^16,17^ and a 56–72% response rate for experimental placebo analgesia^18,19^ when a responder was defined as a participant who reported a change in pain in the expected direction after placebo treatment. Hence, the majority of studies reporting the percentage of placebo responders define a placebo responder as a patient or a participant in an experimental study that reports *any* pain reduction measured on a VAS or numerical rating scale (NRS) after placebo treatment, regardless of the magnitude of the pain reduction^12^.

Here, data from a large experimental placebo analgesic study performed in healthy volunteers^20^ were used to test the assumption that placebo responders can be separated from nonresponders by cutoff values for both absolute and relative percentage changes in pain measured on a 100-mm VAS. Data from 296 participants (179 females) were included. Thermal pain stimulation was performed in two pretests and three posttests, and the temperature for pain stimulation needed to evoke a rating of 60 on a 100-mm computerized visual analog scale was individually determined for each participant in a calibration phase before the pretests. Placebo administration (placebo cream) together with information that the cream was a painkiller was performed after the pretests. The design consisted of a double-blind procedure in the placebo group, whereas the control group received no cream but experienced the same pain procedure as the placebo group.

Unsupervised cluster analysis was used to classify responders and nonresponders, and the classification was tested with different cutoffs in a supervised machine learning algorithm based on logistic regression. If a placebo analgesic manipulation produces either responders or nonresponders, an unsupervised cluster analysis should result in two clusters. Since placebo analgesia is dependent on a reduction in pain reports measured on the same scales as clinical pain, it was hypothesized that reductions in pain after placebo treatment should have the same magnitude as pain reduction after clinical treatment^7,10,11^ to refer to the response as “analgesia”. Consistent with the predictions, the cluster analysis revealed two distinct clusters in the data separated by significant differences in pain change after placebo treatment. Furthermore, the machine learning classification suggested cutoff values for placebo analgesic responses close to the suggested cutoffs for acute clinical pain. The assumption that pain reporting after placebo administration results in a two-cluster solution was supported by testing pain change data from three previously published studies from our lab^21–23^. Consequently, it is suggested that the statistical approach in the present study could be used as an alternative for defining placebo responders in both clinical and experimental studies where patient self-report is the main outcome.

## Results

### Group differences

A comparison of pretest and posttest values in the placebo group showed a significant mean decrease in pain intensity of 16.76 VAS points (95% CI [13.94–19.59]) after placebo manipulations consisting of verbal information and application of a placebo cream (t (145) = 11.72, *p* < .001). The mean values of the two pretests were 58.24 (95% CI [55.67–61.11]), and the mean pain intensity in the posttest was 41.48 VAS points (95% CI [38.39–45.02]) on the 100-mm VAS scale. In contrast, in the control group that received no manipulations during the pain stimulation procedure, the mean decrease in pain was significant (t (124) = 3.04, *p* = .003), with a 3.26 VAS points reduction (95% CI [1.03–5.39]) from the pretest to the posttest. When comparing the placebo group and the control group, a significantly larger pain reduction was observed in the placebo group (t (294) =  − 7.85, *p* < .001). Hence, the design produced a statistically significant placebo analgesic effect.

### Cluster analysis

**Figure 1:**
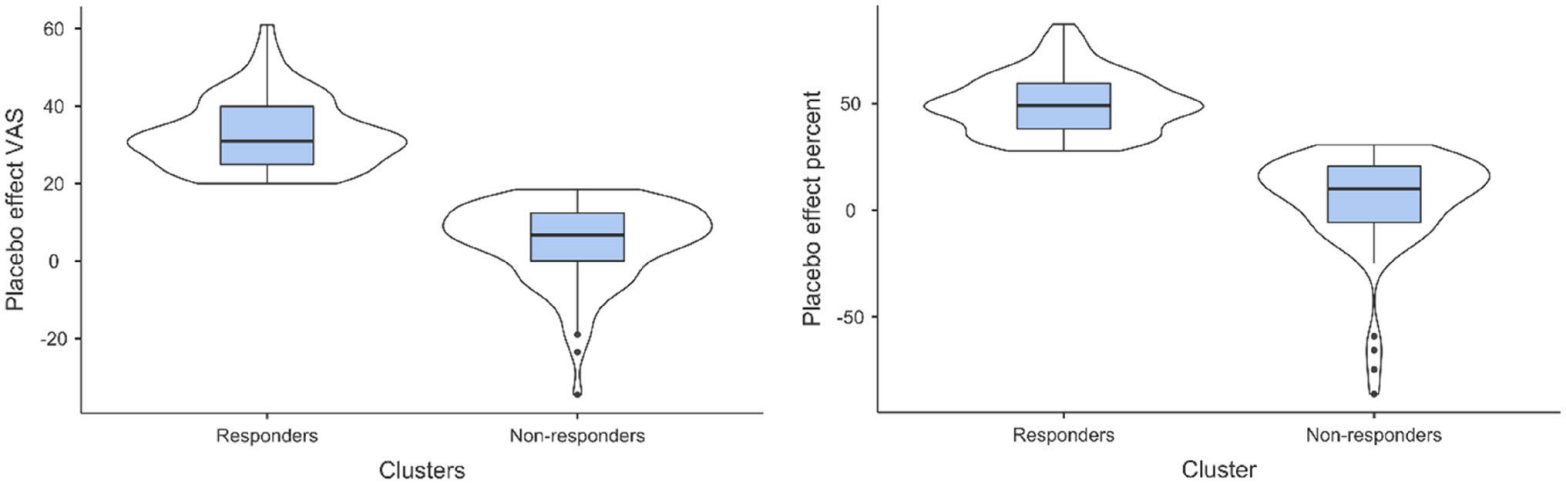
Box and violin plots showing the density of placebo responders versus nonresponders.

**Figure 2:**
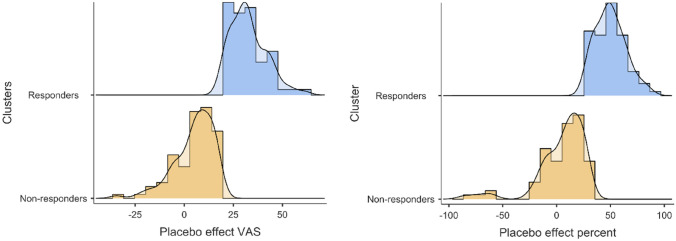
Histograms showing the distribution of responders and nonresponders classified by the two-step cluster analysis.

A two-step unsupervised cluster analysis was performed on the VAS change scores for the mean of the two pretests and the last posttest (pretests–last posttest). Before performing the two-step cluster analysis, the order of the participants in the data file was randomized in order to reduce order-effects. In the placebo group, the analysis revealed two distinct clusters (Fig. 1) with means of 32.68 (95% CI [30.43–34.91]) and 5.16 (95% CI [2.98–7.33]), medians of 31 and 7.5, and modes of 29.5 and 3 for changes in VAS pain ratings. The cluster quality was good, with an average silhouette measure of .7. The two clusters were termed placebo responders (N = 65, 42%) and nonresponders (n = 90, 58%), respectively. The distribution of changes in pain for the responders and nonresponders is shown in Fig. 2, and the mean difference in pain changes between the responders and nonresponders was significant (t (153) = 17.19, *p* < .001). The effect size was very large^24^ based on the mean difference between the responders and the nonresponders (32.68–5.16/SD 16.70 = 1.65; Cohen's *d*). To test the stability of the results from the two-step cluster analysis, the order of the sample was randomized again and split into halves and then run in two separate samples. When splitting the sample, the results were identical to the initial analysis with two clusters and a silhouette measure of .7. The cluster solution with two clusters was additionally tested in a supervised *k*-means cluster analysis. The *k*-means analysis showed that a two-cluster solution produced the same number of responders (N = 65), and nonresponders (N = 90), the two clusters had final cluster centers of 32.68 and 4.88, and the ANOVA test for differences in cluster means was significant (F (1, 153) = 295.54, *p* < .001).

When performing the two-step cluster analytic procedure on data from the control group, four clusters emerged with good quality of the clusters (.7) determined by the average silhouette measure (cluster 1, N = 23, mean = 20.8; cluster 2, N = 42, mean = 8.83; cluster 3, N = 50, mean =  − .09; cluster 4, N = 26, mean =  − 13.13).

### Validation of the two-cluster solution for placebo responding

The unsupervised cluster analysis for classification was additionally tested on data from the placebo arms and/or conditions in published studies with smaller samples from our lab^21–23^. The two-step cluster analysis classified the pain change data in two-cluster solutions in all three datasets, with silhouette measures .7, .7, and .6, respectively. Thus, the classification produced cluster solutions of good quality, and the assumption that self-reported responses after placebo administration can be classified in responders and non-responders was supported. See Supplemental information for details about the validation of the cluster solution.

### Determination of cutoff values by machine learning

The two categories (placebo responders and nonresponders) derived from the cluster analysis in the placebo group were used as dependent variables in a machine learning algorithm based on logistic regression. Values for pain change ranging from 0 to 50 for percentage change and change values from 0 to 25 for absolute VAS change were tested as possible cutoff values. The false discovery rate (FDR) was used to assess the predictive ability for each of the selected cutoff values, and q ≤ 5% was chosen as the FDR criterion for a valid cutoff that could separate responders from nonresponders. The optimal cutoff values (Table 1) for a VAS change were 18–20. These values produced identical area under the curve (AUC) values (.99) and showed high accuracy (99.4%) for the prediction of the placebo responders identified by the cluster analysis. Furthermore, low (0–1%) FDR values for both false classification of responders and nonresponders suggested a highly precise prediction. A VAS change value of 17 also reached the FDR criterion but with somewhat lower AUC and accuracy values. In summary, absolute VAS changes in the range between 17 and 20 points could separate responders from nonresponders within the 5% FDR criterion. In comparison, a cutoff based on any or a small change in pain (≥ 0) showed a predictive value close to chance level (AUC = .61), whereas a cutoff based on a large VAS change score (≥ 25) correctly classified all the nonresponders but incorrectly classified 17% of the responders as nonresponders. Thus, using higher cutoff values may increase the false negative rate. When using different cutoffs for percentage change values (0 to 50% change in VAS ratings), the optimal cutoffs were 27–28% change (AUC = .99, accuracy = 99.3%) with FDR values of 0–1% (Table 1). Percentage changes between 25 and 30 all passed the 5% FDR criterion but with lower AUC (.96–.97) and accuracy values (97.3–97.9%). Calculation of the Youden index^25^ was additionally performed to compare cutoffs from the logistic machine learning analysis with a widely used method for this purpose^26^. Generally, the machine learning procedure and the Youden method gave similar cutoff values (see Table 1).

**Table 1:** Cutoff values from logistic regression machine learning.

VAS absolute change	AUC	Accuracy %	FDR responder %	FDR non-responder %	Youden index
0	.61	55.5	52	36	.27
5	.66	65.8	45	0	.40
10	.79	78.7	33	0	.62
15	.9	91	18	0	.83
16	.95	95.5	10	0	.91
**17**	**.98**	**98.7**	**3**	**0**	**.97**
**18**	**.99**	**99.4**	**1**	**0**	**.98**
**19**	**.99**	**99.4**	**0**	**1**	**1.0**
**20**	**.99**	**99.4**	**0**	**1**	**1.0**
21	.94	96.8	0	5	.94
22	.92	95.5	0	7	.91
23	.9	94.2	0	9	.88
24	.86	91.6	0	13	.82
25	.84	88.4	0	17	.74

VAS % change	AUC	Accuracy %	FDR responder %	FDR non-responder %	Youden index
0	.64	69.2	36	0	.29
5	.69	74	32	0	.38
10	.77	80.1	27	0	.50
15	.83	86	20	0	.62
20	.91	92.5	12	0	.74
25	.96	97.3	5	0	.88
**26**	**.97**	**97.9**	**4**	**0**	**.89**
**27**	**.99**	**99.3**	**1**	**0**	**.93**
**28**	**.99**	**99.3**	**0**	**1**	**.96**
**29**	**.97**	**97.9**	**0**	**4**	**.94**
**30**	**.97**	**97.9**	**0**	**4**	**.95**
31	.96	97.3	0	6	.95
32	.94	95.9	0	8	.92
33	.93	93.8	0	12	.89
34	.9	91.8	0	15	.85
35	.89	91.1	0	16	.84
40	.84	84.9	0	25	.72
45	.81	81.5	0	29	.66
50	.72	72.6	0	37	.49

The VAS absolute change is the absolute change in VAS pain intensity ratings from the pretests to the posttest. The VAS percent change is the percentage change in VAS pain intensity ratings from the pretests to the posttest. Bold type indicates q ≤ 5%.

*VAS* visual analog scale, *AUC* area under the curve, *FDR* false-discovery rate.

## Discussion

The gold standard for the determination of a placebo effect in experimental designs is the observation of statistically significant differences between one or more groups receiving placebo treatment and a group receiving the same procedure but with no treatment^27^. Hence, this definition is based on observed differences in measures of central tendencies, most often mean differences, regardless of the magnitude of the change; when statistical significance is used as the only criterion, this might not account for the physiological and psychological experience of the stimulation or whether analgesia is induced.

The results from the present study and studies on cutoffs for clinical pain^7–11^ suggest that small, albeit statistically significant, changes in pain after treatment may have no practical or clinical relevance. Furthermore, when using any pain change in the desired direction as the definition of placebo responders, the present data showed that the accuracy was close to chance. This is a problem for the interpretation of experimental studies on placebo analgesia with no criterion for defining when changes in pain magnitude are sufficient to be considered meaningful decreases in pain perception. However, studies with small but significant changes in pain may still provide important information about mechanisms of placebo-induced changes in physiological systems related to pain perception^21,28^ and how contextual and psychological factors associated with placebo administration may affect treatment or experimental outcomes; see^2,29,30^ for an overview. Nonetheless, the term “analgesia” refers to the relief of pain without the loss of consciousness, and studies making inferences about placebo analgesia should at least display changes in pain ratings large enough to be recognized as reduced pain intensity that are perceived as such.

The results from the present study showed a significant difference in mean pain change between the placebo group and the control group. Nonetheless, the cluster analysis revealed that one of the clusters in the control group data had a pain reduction that could be classified as placebo responses according to the cutoff values found in the present study if it had been observed in the placebo group. Moreover, the second cluster in the control group had a mean pain change of 8.83, which is comparable to placebo induced pain changes in several previous studies^5,12,31,32^. These findings suggests that relatively large changes in experimental pain reports could occur in the absence of a treatment and be caused by factors such as reporting biases, individual differences in emotional responses to the pain stimulation, and statistical regression to the mean^33–35^. Thus, the possibility exist that parts of the improvement observed in the placebo group would also have occurred without the placebo treatment^36^.

The present study is probably the first to provide cutoff values for experimental placebo analgesia. The optimal cutoff values of 18–20 mm for absolute VAS changes and 27–28% changes are not surprising given findings in studies on clinical pain^7–11^. However, the cutoff suggested by Todd et al.^7^ of a 13-mm change for acute pain was too liberal in the present data, as shown by both the machine learning analysis and the Youden index.

Nonetheless, the cutoffs found in the present study were based on a specific procedure for placebo treatment and pain stimulation, and different procedures might provide different values. A limitation of the present data is that the pain measurement did not include a qualitative measure for rating the perceived pain^37^ after placebo treatment as “much less", "little less", "the same", etc. Hence, minimally clinically important difference (MCID) values could not be computed. On the other hand, the present results are in line with suggested cutoff values found in studies on clinical pain, and the pain stimulation used here is one of the most common methods in experimental pain studies^38^. The use of cutoff values for pain changes to perform responder analyses has been recommended for clinical pain trials with the reasoning that statistical mean differences might not capture the experienced effect of pain reductions in most patients^39–41^. Thus, the definition of placebo responders versus nonresponders based on cutoffs could be a beneficial add-on for analyses for both clinical and experimental studies. However, the terms “placebo responders and nonresponders” does not imply that participants who shows a positive response to placebo administration in e.g., a pain study will be placebo responders in other situations. Placebo responding and placebo nonresponding are probably not consistent traits^14,42^, but depend on the individual learning history of the participants^43^, level of emotional activation during treatment^44^, and genetic factors^20,45^ associated with the sensory modality in which the placebo treatment is used.

The magnitude and impact of placebo analgesic effects in clinical trials and experimental studies have been debated, e.g.,^12,31,46,47^. Nonetheless, these meta-analyses have shown that placebo administration has a statistical impact on self-reported pain. Furthermore, Zunhammer et al.^48,49^ found in meta-analyses that placebo administration has minor effects on the neurologic pain signature^50^ but moderate effects on pain reports, which suggests that the measurement and analysis of pain reports are crucial for determining the magnitude of placebo analgesic effects. Nevertheless, most meta-analyses have not analyzed whether the observed effects of placebos had a clinically meaningful impact. To date, the only meta-analysis that tested clinical significance in relation to placebo analgesia found that the placebo analgesic effect was higher in patients than in healthy controls in terms of clinically significant reductions in pain when using the reduction criteria of ≥ 13 mm^7^ on a 0–100 VAS or NRS scale^12^. Interestingly, meta-analyses that have used effect sizes as the main outcome measure have shown the opposite finding: the placebo effect is larger in healthy controls than in patients^31,32^. Taken together, analyses of placebo effects in future studies should preferably include assessments of both statistical and clinical significance, at least in studies making inferences about patient-reported outcomes such as pain^51^.

The present study shows that placebo responders can be separated from nonresponders with a straightforward procedure based on unsupervised cluster analysis and cutoffs selected by logistic regression implemented in machine learning. These statistical tools are available in several statistical software packages and could easily be used by scientists with some experience in statistics. Moreover, the machine learning application used here can be replaced by an ROC curve analysis providing data for sensitivity and specificity^52^ that is implemented in most statistical programs or software specialized for finding optimal cutoff points^53^. However, the latter does require that the patients/participants be classified as either responders or nonresponders before the analysis to find the optimal cutoff is performed. Thus, in cases where there is no diagnostic information or clinical classification, an unsupervised cluster analysis may help the categorization process without inducing bias associated with manual human classification. The two-cluster classification of the placebo responses of the present study was replicated in other data from our lab. However, other types of experimental designs may provide more complex classification of responses^54^. In data where more than two clusters are found, the logistic regression and eventually the Youden index must be replaced with classification analyses that handle multiclass data such as support vector machines or similar methods. An advantage of machine learning algorithms for classification over standard statistical methods is that these applications provide additional statistics, such as FDR values, compared to other analysis such as logistic regression. Furthermore, the flexibility in model specifications, such as validation schemes of the selected model, provides better control over the accuracy and error rate compared to standard statistical methods.

## Methods

Data from 296 healthy subjects who participated in an experimental study on genetic factors in placebo analgesia^20^ were used for the present analyses. The characteristics of the sample are described in detail in previous publications^20,55^.

The participants were randomized into three groups: (1) a placebo group (N = 155) that received a moisturizing cream with no analgesic properties (E-45; Crookes Healthcare, Nottingham, United Kingdom), (2) a natural history group (N = 141) that received no treatment during the procedure, or (3) a lidocaine-prilocaine cream group (N = 31) that received a local anesthetic cream (Emla; AstraZeneca, Oslo, Norway)^20^. Thus, the study included a total of 327 healthy participants with a mean age of 23 years (SD = 3.3). A dose of 3 g of Emla or E-45 was used for each participant. The group receiving the anesthetic cream was used in the design to ensure blinding of the experimenters, and these data were not used in the analyses^20,55^. The experiment was run in a double-blind manner for the groups where a cream was applied, but there was no blinding in the natural history group. Randomization was performed before the start of the experiment. The participants were allocated to the different groups based on their participant number. The participant numbers and group allocations were randomized by using the online web service https://www.random.org/lists/. Thirty-one of the participants were randomized into the lidocaine-prilocaine cream group. Hence, data from 296 participants were included in the final analyses^20^. The study was reviewed and approved by the Regional Committee for Medical and Health Research Ethics, Region Northern Norway (project number 23430), and the study was performed in accordance with The Declaration of Helsinki. All participants signed an informed consent before participation. In the consent form, they stated that they had no history of ongoing disease or any history of serious disease.

### Design and stimuli

The study was designed as a repeated measures design with a calibration phase for determination of temperatures that invoked pain corresponding to 60 mm on a 100-mm computerized visual analog scale (VAS) scale. Thermal stimuli to the left underarm were delivered by a computer-controlled thermode (Pathway, Medoc, Israel) to induce heat pain. To ensure an equal pain level across participants at the start of the experiment, a calibration procedure was performed. To approximate the stimulus intensity needed to produce a rating of 60 on the VAS, Stevens power equation^56^ was used to produce a stimulus–response function for estimation of the individual target temperature of the thermode. After the calibration phase, the pretest consisting of the presentation of two pain stimuli was performed. The duration of the stimulations in the pretests and posttests was 10 s from when the thermode reached the calibrated target temperature (43–47 °C) until the start of the return to baseline at 32 °C^20,55^. The temperature of the thermode increased/decreased by 10 °C/s. The interval between the two pretests was 30 s. The two posttests had the same temperature, duration, and intervals as the pretests^20,55^. Directly after the pretests, information about the treatment was provided to the participants allocated to the groups that received either placebo or Emla^20,55^. The participants in the placebo group were told “The cream that will be applied to your arm reduces pain, the substance in the cream is used as a local anesthetic in many pain-reducing remedies and is effective in the treatment of heat pain.” The participants in the natural history group received no application of cream and were told that they could relax in the break between the pretests and the posttests^20,55^. The experimental procedure had a total duration of approximately 45 min, which included saliva sampling for genetic analyses and measurements of blood pressure and subjective stress.

### Statistical analyses

A two-step cluster analysis in SPSS v. 26 (SPSS, IBM, USA) was used to classify changes in VAS pain intensity from the pretest to the posttest. The order of the participants in the datafile was randomized by the *randperm* function in MATLAB (MATLAB v.R2019b, Sweden). The cluster analysis was set to automatically determine the optimal number of clusters by using Schwarz’s Bayesian criterion^57^ and log-likelihood as the distance measure https://www.ibm.com/support/knowledgecenter/SSLVMB_24.0.0/spss/base/idh_twostep_main.html . The quality of the cluster analysis was measured by the silhouette coefficient, which is a measure of both cohesion and separation of the suggested clusters. MATLAB Classification Learner (MATLAB, Sweden; https://se.mathworks.com/help/stats/classificationlearner-app.html) with cross-validation (fivefolds) was used for the prediction of cutoff values from the two-step cluster analysis. The logistic regression classifier was used for classification of the selected possible cutoffs for the absolute VAS change and percentage change between the pre- and posttests. The possible cutoffs were tested separately. Predictive values were determined from ROC curves providing area under the curve (AUC) values and a confusion matrix that provided false discovery rate (FDR) values. The accuracy of the predictor is in the Classification Learner calculated from the cross-validation. The Youden index was calculated in SPSS based on a script (https://www.ibm.com/support/pages/can-spss-produce-youdens-index). Group comparisons were performed with two-tailed t-tests. *p* values < .05 were considered significant.

## Supplementary Information

Supplementary Information.

## Data Availability

All data used in the analyses for this article can be found at https://dataverse.no/.
