# Supplementary material for: Cutoff criteria for the placebo response: a cluster and machine learning analysis of placebo analgesia
Source: Sci Rep. 2021 Sep 28;11:19205. doi: 10.1038/s41598-021-98874-0 (PMC8479132; doi:10.1038/s41598-021-98874-0)
Supplement: Supplementary file 1 — Supplementary Information. [file 41598_2021_98874_MOESM1_ESM.pdf]

Supplementary information for the article "Cutoff criteria for the placebo response: a cluster and machine learning analysis of placebo analgesia".

Validation of the two-cluster classification of placebo analgesic responses

Data for change in pain reports after placebo administration were obtained from three published studies from the Pain Lab at the Department of Psychology, UiT The Arctic University of Norway [1-3]. Data from the placebo arms from these studies were used to replicate the findings from the cluster analysis in the main dataset with a larger sample [4]. All three studies employed thermal heat pain as pain stimuli, were run double-blind, and all studies used a repeated measures design for pain stimulation. Two-step unsupervised cluster analysis was performed on all three datasets of the placebo groups/conditions separately. The cluster analysis was set to automatically determine the optimal number of clusters, by using the Schwarz's Bayesian Criterion [5], and Log-Likelihood as distance measure in SPSS https://www.ibm.com/support/knowledgecenter/SSLVMB_24.0.0/spss/base/idh_twostep_main.html . The quality of the cluster analysis was measured by the silhouette coefficient. Before running the two-step cluster analysis, the order of the participants in the datafile was randomized by the *randperm* function in MATLAB. Data used for the analyses can be found at https://dataverse.no . All graphs were created in JAMOVI [6]. Supplemental Table 1 shows comparisons between the clusters (responders vs nonresponders) from the included data sets.

Replication dataset 1

In Aslaksen & Flaten [1], sixty-three healthy volunteers (32 females) participated in two experimental conditions (within-subjects) performed on separate days, one day for the placebo condition and one day for the natural history condition. In the placebo condition, the participants received placebo capsules containing lactose together with information that the capsules were potent painkillers, whereas in the natural history condition, the same pain procedure (one pretest, four posttests) was performed but without placebo administration. The temperature of the thermode was 46°C (240 seconds duration). The pain data showed a significant ($p < .05$) placebo effect on mean pain intensity of (56.37 – 49.10) 7.27 VAS points on a 0-100 scale.

The cluster analysis revealed two clusters with an average silhouette coefficient of .7.

Supplemental figure 1.

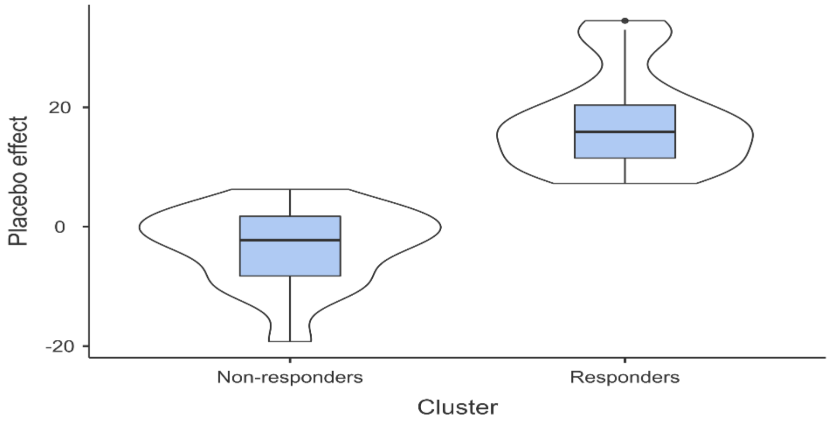

Supplemental figure 1. Boxplots/violin plots showing the two groups based on the two-step cluster analysis. The Y-axis show the placebo effect (pretest VAS – posttest VAS). Nonresponders, N = 31; Responders N = 32. Data from Aslaksen & Flaten [1].

Supplemental figure 2

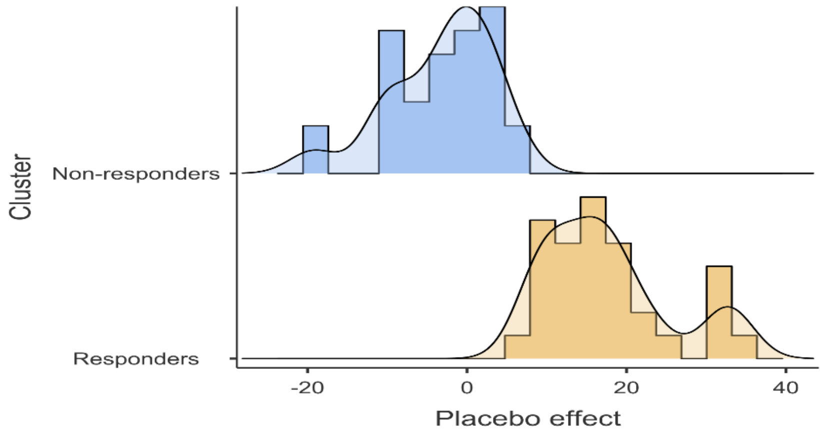

Supplemental figure 2. Histograms/density graphs showing the distributions in the two clusters. Nonresponders, N = 31; Responders N = 32. Data from Aslaksen & Flaten [1].

Replication dataset 2

In Aslaksen, Vasylenko & Fagerlund [2] seventy-five healthy volunteers participated in a study that tested the effect of Transcranial Direct Current Stimulation (tDCS) on thermal heat pain. Pretests, pain stimulations under treatment, and posttests with three different stimulation temperatures for 20 seconds duration (43°C, 45°C, 47°C) were performed. The placebo group (N = 32, 19 females) received sham tDCS, whereas the active tDCS group received 2mA tDCS stimulation, and the natural history group got the same pain stimuli but no tDCS montage. The placebo group had a reduction in pain reports of (62.81 – 56.89) 5.92 VAS

points on a 0-100 scale in the 47°C condition ($p < .05$). The cluster analysis revealed two clusters with an average silhouette coefficient of .7.

Supplemental figure 3.

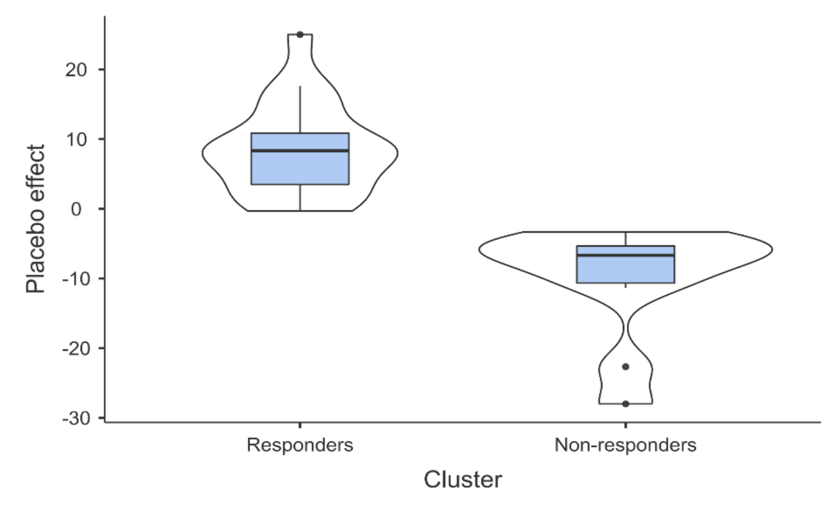

Supplemental figure 3. Boxplots/violin plots showing the two groups based on the two-step cluster analysis. The Y-axis is the placebo effect (pretest VAS – posttest VAS). Responders, N = 19; nonresponders N = 11. Data from Aslaksen, Vasylenko & Fagerlund [2].

Supplemental figure 4.

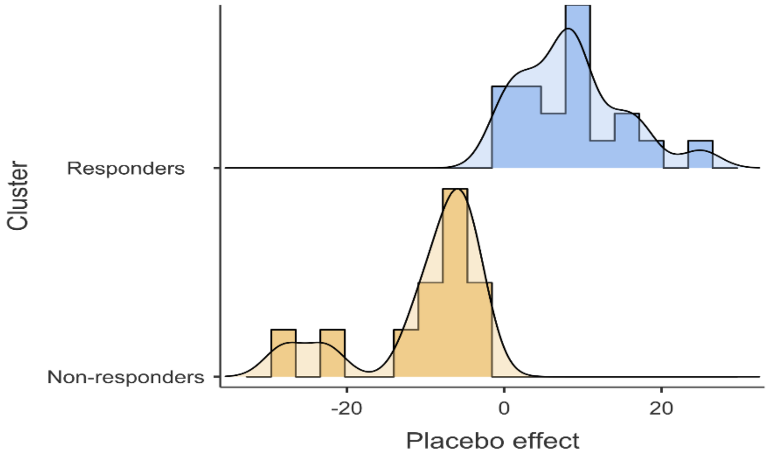

Supplemental figure 4. Histograms/density graphs showing the distributions in the two clusters. Responders, N = 19; nonresponders N = 11. Data from Aslaksen, Vasylenko & Fagerlund [2].

Supplemental Table 1

Study	Responders				Nonresponders				t	p	*d*
	Mean	SD	Median	IQR	Mean	SD	Median	IQR			
Aslaksen & Flaten [1]	17.5	7.96	15.9	8.88	-3.31	6.51	-2.25	10	11.3	<.001	2.86
Aslaksen, Vasylenko & Fagerlund [2]	8.49	6.62	8.33	7.33	-10.1	7.99	-6.67	5.33	6.87	<.001	2.6
Aslaksen, Zwarg, Eilertsen, Gorecka & Bjorkedal [3]	13.9	15	8.5	15.8	-12.8	12.3	-7.75	11.8	4.91	<.001	1.98

Supplemental Table 1. Comparisons between the clusters (responders vs nonresponders) from the included data sets. SD = Standard Deviation. IQR = Interquartile range. *d* = Cohen's *d*.

Replication dataset 3

In Aslaksen, P. M., Zwarg, M. L., Eilertsen, H. H., Gorecka, M. M. & Bjorkedal, E [3] 142 healthy volunteers (73 females) were randomized to six groups whereas two of the groups were a placebo group (N = 23, 12 females) and a natural history group (N = 25, 13 females). Pain was induced at 48°C in a pretest and two posttests with 15 seconds duration at the target temperature. In the placebo group, participants received placebo cream together with information that the cream was a painkiller, whereas in the natural history group the same pain induction was performed but without placebo administration. The results showed no significant effect of the placebo when comparing the pretest with the last posttest with a pain reduction of (47.89 – 47.06) .83 VAS points after placebo administration on a 0-100 scale. The cluster analysis revealed two clusters with an average silhouette coefficient of .6.

Supplemental figure 5.

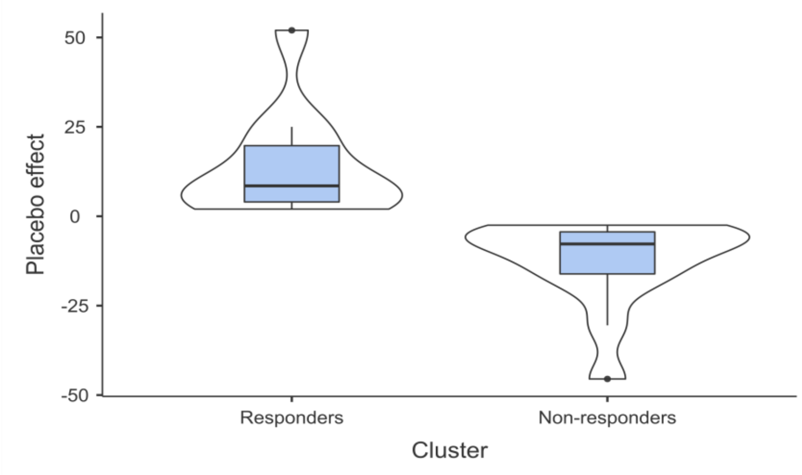

Supplemental figure 5. Boxplots/violin plots showing the two groups based on the two-step cluster analysis. The Y-axis is the placebo effect (pretest VAS – posttest VAS). Responders, N = 11; nonresponders N = 14. Data from Aslaksen, Zwarg, Eilertsen, Gorecka, Bjørkedal [3].

Supplemental figure 6.

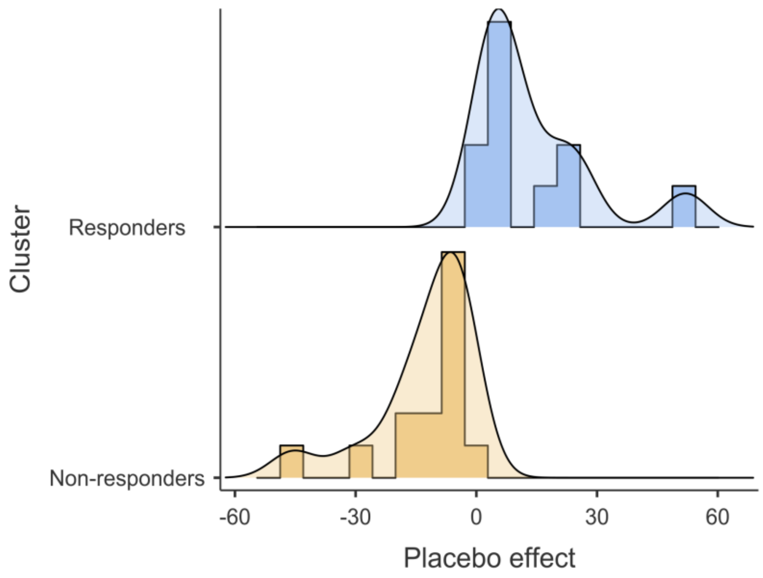

Supplemental figure 6. Histograms/density graphs showing the distributions in the two clusters. Responders, N = 11; nonresponders N = 14. Data from Aslaksen, Zwarg, Eilertsen, Gorecka, Bjørkedal [3].

Supplemental references

1 Aslaksen, P. M. & Flaten, M. A. The roles of physiological and subjective stress in the effectiveness of a placebo on experimentally induced pain. *Psychosom Med* **70**, 811-818, doi:10.1097/PSY.0b013e31818105ed (2008).

2 Aslaksen, P. M., Vasylenko, O. & Fagerlund, A. J. The effect of transcranial direct current stimulation on experimentally induced heat pain. *Exp Brain Res* **232**, 1865-1873, doi:10.1007/s00221-014-3878-0 (2014).

3 Aslaksen, P. M., Zwarg, M. L., Eilertsen, H. H., Gorecka, M. M. & Bjorkedal, E. Opposite effects of the same drug: reversal of topical analgesia by nocebo information. *Pain* **156**, 39-46, doi:10.1016/j.pain.0000000000000004 (2015).

4 Aslaksen, P. M., Forsberg, J. T. & Gjerstad, J. The opioid receptor mu 1 (OPRM1) rs1799971 and catechol-O-methyltransferase (COMT) rs4680 as genetic markers for placebo analgesia. *Pain* **159**, 2585-2592, doi:10.1097/j.pain.0000000000001370 (2018).

5 Schwarz, G. Estimating the dimension of a model. *The annals of statistics* **6**, 461-464 (1978).

6 Project, T. J. Jamovi (Version 1.6) [Computer Software]. (2020).
